# Reusable respirators as personal protective equipment during ENT surgery

**DOI:** 10.1017/S0022215120001346

**Published:** 2020-07-01

**Authors:** B Patel, J C Hardman, W Yang, A Robson, G Putnam, A George, V Paleri

**Affiliations:** 1 Head and Neck Unit, Royal Marsden NHS Foundation Trust, London, UK; 2 Department of ENT, North Cumbria Integrated Care NHS Foundation Trust, Carlisle, UK; 3 Department of Head and Neck, University Hospitals of North Midlands NHS Trust, Stoke-on-Trent, UK

**Keywords:** COVID-19, Personal Protective Equipment, Cost-Benefit Analysis, Communication

## Abstract

**Background:**

Robust personal protective equipment is essential in preventing the transmission of coronavirus disease 2019 to head and neck surgeons who are routinely involved in aerosol generating procedures.

**Objective:**

This paper describes the collective experience, across 3 institutes, of using a reusable half-face respirator in 72 head and neck surgery cases.

**Method:**

Cost analysis was performed to demonstrate the financial implications of using a reusable respirator compared to single-use filtering facepiece code 3 masks.

**Conclusion:**

The reusable respirator is a cost-effective alternative to disposable filtering facepiece code 3 respirators. Supplying reusable respirators to individual staff members may increase the likelihood of them having appropriate personal protective equipment during their clinical duties.

## Introduction

Otorhinolaryngologists and head and neck surgeons are at particular risk of contagion from highly communicable diseases, including coronavirus disease 2019 (Covid-19), owing to their involvement in aerosol generating procedures in the upper aero-digestive tract.^1^ More than half of patients with Covid-19 may be asymptomatic carriers of the severe acute respiratory syndrome coronavirus-2 virus.^2^ As such, ENT UK recommends robust personal protective equipment (PPE) for all patients during the current pandemic to reduce the chance of Covid-19 transmission to clinicians.^3^ This currently includes filtering facepiece code 3 (FFP3) masks, which achieve a 100-fold reduction in exposure and a minimum filter efficiency of 99 per cent when correctly fitted.^4^

Here, we describe the combined surgeon experience, across 3 institutions, of using a reusable non-powered respirator during 71 head and neck procedures. We also outline the cost implications of using reusable respirators compared to disposable FFP3 masks.

## Technical notes regarding device

The Sundström SR 100 respirator (Sundström, Lagan, Sweden) is a reusable half-face mask device, used primarily in industrial settings, to filter gas and vapour particulates. The accompanying SR 510 P3 filter and SR 221 pre-filter enable the capture of particulates equivalent to existing FFP3 masks. The P3 filter can be used for up to two weeks before it needs to be changed, while the SR 221 pre-filter should be changed weekly.^5^ Two exhalation valves keep the exhalation resistance low and help to prevent humidity build up within the mask, improving comfort during extended use.

**Fig. 1.**
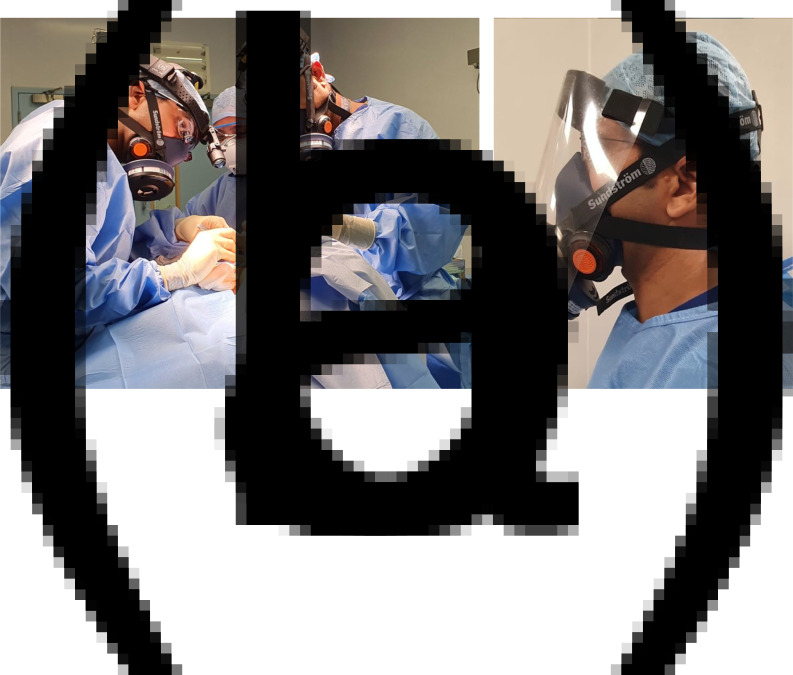
Respirator worn with (a) loupes and headlight, and (b) visor for eye protection.

The body of the mask is made of flexible silicone and comes in three sizes (small to medium, medium to large and large to extra-large), all with adjustable straps, allowing the respirator to accommodate a variety of head shapes. In order to ensure appropriate personal protection, the mask should be ‘fit-tested’ and used in conjunction with suitable eye protection. Users should also perform an additional ‘fit-check’ each time the respirator is used. This is performed by manually occluding the P3 filter and inhaling sharply; correctly fitted masks will not allow air flow, and there will be no air leak at the edges of the respirator. The half-face design of the Sundström SR 100 respirator allows it to be used alongside full face visors and goggles, as well as surgical loupes commonly used in head and neck surgery (Figure 1).

Between patients, the external surfaces of the mask should be cleaned with disinfectant wipes for a minimum of 15 seconds. Suitable wipes include Clinell Universal Wipes (Gama Healthcare, Watford, UK) or Virusolve Wipes (Amity International, Barnsley, UK), both of which have virucidal properties.

## Clinical use

The authors have experience using the Sundström SR 100 respirator for a variety of head and neck procedures lasting up to 8 hours; these include endoscopy with or without biopsy (*n* = 32), speaking valve changes (*n* = 15), neck dissection (*n* = 6), thyroidectomy (*n* = 6), lymph node biopsy (*n* = 4), surgical tracheostomy and tracheostomy tube changes (*n* = 3), wide local excision of intra-oral lesions (*n* = 3), laser cordectomy (*n* = 1), and salvage segmental mandibulectomy (*n* = 1). The respirator allowed the surgeons to operate uninterrupted for all procedures, except the mandibulectomy case, where a single comfort break was taken during the 8-hour procedure.

## Cost analysis

The cost per unit for a reusable respirator, supplied with an appropriate filter, is approximately £34. Replacement filters cost £0.29. Alternatively, disposable FFP3 masks cost approximately £3.40 per unit and are recommended to be changed for each new patient. In addition, disposable FFP3 masks should not be used continuously for more than 1 hour; therefore, multiple FFP3 masks may be required for surgical cases lasting more than 1 hour.^1^ Table 1 demonstrates potential savings of £150 by using the reusable respirator for one month. The cost of acquiring a respirator is recovered after it is used for 10 patients.

**Table 1.** Costs associated with using a respirator compared to disposable face masks over a month

Costs	SR 100 Respirator (£)	Disposable FFP3 mask (£)
Initial outlay	33.74	–
Recurring costs		
– SR 221 pre-filter (replaced weekly)	0.29	–
– SR 510 P3 R particle filter (replaced every 2 weeks)	6.98	–
Per patient cost		
– Wipes	0.09	–
– Mask	–	3.40
Weekly cost		
– (3 patients per day, 5 days a week)	5.13	51.00
Cumulative costs over 1st month		
– Week 0	34	–
– Week 1	35	51
– Week 2	44	102
– Week 3	46	153
– Week 4	54	204

FFP3 = filtering facepiece code 3

## Limitations

The authors found that the principal limitation of prolonged mask use was the pressure elicited over the nasal dorsum. The use of a hydrocolloid dressing, such as DuoDerm (ConvaTec, Deeside, Wales, UK) or Comfeel Plus (Coloplast, Humlebæk, Denmark), was effective in improving comfort for subsequent cases, though it should be noted that fit testing and checking should be performed with these dressings in situ, prior to use in the clinical environment.^6^

The large filter design allows comfortable inhalation with low airflow resistance during normal use. However, the surgeon must be mindful of the projection of the filter unit towards the surgical field when operating. Inattention to this projection risks the inadvertent contamination of surgical instruments and equipment. In addition, the respirators, as with other FFP3 masks, are only intended to protect the user. The exhalation valves do not filter exhaled air; therefore, as with other FFP3 masks, Covid-19-positive mask users can transmit the virus to people around them during use.

Finally, and most significantly, larger respirators, such as the Sundström SR 100, notably impede voice projection when compared to lighter-weight, single-use alternatives. Clear communication between surgeons, the scrub team and other operating theatre staff is essential for safe operating. Before each procedure, the authors ensured that all members of the surgical team were clearly briefed about anticipated steps and potential complexities. Intra-operatively, background noise was kept to a minimum, and verbal communication was kept short and focused to reduce the chances of misinterpretation.

## Conclusions

The availability and use of appropriate PPE is of particular significance during the current Covid-19 pandemic, to ensure the safety and welfare of all members of the surgical team. The FFP3 standard masks are recommended for all at-risk patient encounters in ENT practice, but these may be limited in availability in some areas. Guidelines recommend the use of a new disposable FFP3 mask for each surgical case, which is costly and generates excess clinical waste.^1^ A reusable respirator is a safe and convenient solution that can be supplied individually to staff members. It is cost effective after only a short period and may increase the likelihood of the mask user having appropriate personal protection during their clinical duties.
